# Influence of water on the properties of an Au/Mpy/Pd metal/molecule/metal junction

**DOI:** 10.3762/bjnano.2.44

**Published:** 2011-07-12

**Authors:** Jan Kučera, Axel Groß

**Affiliations:** 1 Institute of Theoretical Chemistry, Ulm University, D-89069 Ulm, Germany

**Keywords:** density functional calculations, self-assembled monolayers, water adsorption

## Abstract

The geometric and electronic structure of the metal–molecule interface in metal/molecule/metal junctions is of great interest since it affects the functionality of such units in possible nanoelectronic devices. We have investigated the interaction between water and a palladium monolayer of a Au(111)/4-mercaptopyridine/Pd junction by means of DFT calculations. A relatively strong bond between water and the palladium monolayer of the Au/Mpy/Pd complex is observed via a one-fold bond between the oxygen atom of the water molecule and a Pd atom. An isolated H_2_O molecule adsorbs preferentially in a flat-lying geometry on top of a palladium atom that is at the same time also bound to the nitrogen atom of a Mpy molecule of the underlying self-assembled monolayer. The electronic structure of these Pd atoms is considerably modified which is reflected in a reduced local density of states at the Fermi energy. At higher coverages, water can be arranged in a hexagonal ice-like bilayer structure in analogy to water on bulk metal surfaces, but with a much stronger binding which is dominated by O–Pd bonds.

## Introduction

Recently, an elegant electrochemical method for the metalization of molecular layers assembled on surfaces has been established [1]. Within the procedure, a solution containing metal cations is brought into contact with a self-assembled monolayer (SAM) consisting of organic molecules on a metal substrate, thus forming metal cation/molecule complexes. Then the cationic solution is exchanged with a cation-free electrolyte, and the cation/molecule complexes are reduced under potential control resulting in a metal layer on top of the SAM. The application of this technique led recently to the preparation of various metal/SAM/metal junctions on Au(111) electrodes, involving SAMs formed by 4-mercaptopyridine (Mpy) [1], 4-aminothiophenol (ATP) [2], thiazole [3], or 1,4-dicyanobenzene [4] molecules covered by monolayers of Pd [1], Pt [5], or Rh [6], respectively. In addition, further progress extended the limits of the method towards the preparation of a prototypical Au/Mpy/Pd/Mpy/Pt double decker junction [7].

These achievements can eventually open the way towards the rational design of future bionanoelectronics in which the organic (molecule) and inorganic (metal) units will communicate with each other. Yet, there are many fundamental questions open with respect to the factors that play a crucial role in the preparation, characterization, and operation of metal/SAM/metal junctions. Among those, the elucidation of the microscopic structure of the metal–molecule interface is of particular importance since it influences the functionality of possible devices to a large extent. The knowledge about the metal–molecule contact on an atomic level is still limited because of the considerable complexity of this hybrid system which makes the experimental clarification of microscopic details rather difficult. Here the combination of experimental approaches together with modern methods of quantum chemistry might help to shed light on the microscopic structure of the constituents of the contacts [2,7–9].

The structure of the Pd layers prepared on Au/Mpy and Au/ATP SAMs was recently the subject of several experimental and theoretical studies [7–12]. Experimental ultraviolet photoelectron spectroscopy (UPS) revealed a relatively large reorganization of the valence band of the palladium monolayer with respect to bulk Pd [7–8]. For both the Mpy- and the ATP-SAM the density of states (DOS) of the Pd monolayer was found to be significantly reduced at the Fermi level with the maximum of the DOS shifted to about −1.8 eV below the Fermi energy.

Two possible scenarios have been considered as an explanation for the observed DOS of the palladium layers. Either the DOS might be modified due to the strong SAM–Pd interaction, or additional species from the liquid environment bound to the Pd layer could affect the Pd DOS [7,10,12]. In the case of SAMs formed by ATP molecules, periodic density functional theory (DFT) calculations of the bare Au/ATP/Pd junction, assuming a (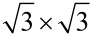) structure of the ATP molecules, were able to reproduce the experimentally observed downshift of the Pd DOS reasonably well [2,12] under the assumption that the amino groups of the ATP molecules become dehydrogenated upon the metalization. The isolated nitrogen atom of the dehydrogenated amino group interacts strongly with three Pd atoms thus causing the strong modification of the DOS.

In the case of the Au/Mpy/Pd junction, on the other hand, the DFT calculations for the bare system only yield a negligible downshift of the DOS [10], in contrast to the experimental results. The nitrogen atom of the Mpy molecule that is part of the aromatic ring interacts directly with only one atom of the Pd layer. The DOS of the Pd atoms not bound to the nitrogen atom remains rather bulk-like such that no significant reduction of the DOS at the Fermi level results. Only upon the consideration of additional adsorbents, such as sulfur, nitrogen, thiolates, amines, or H on the Pd layer, can a downshift of the DOS in agreement with the experiment be obtained in the calculations [12]. However, there is no clear experimental evidence yet with respect to the presence of these adsorbates on the Pd layer. Hence it is fair to say that the reason for the strong downshift of the Pd DOS in the Au/Mpy/Pd junction is still unclear.

The electrochemical metalization of the SAMs occurs in the presence of an aqueous electrolyte. In order to obtain a complete understanding of the factors influencing the geometric and electronic structure of the Au/Mpy/Pd junctions, it is important to clarify the role of the water–palladium interaction on the properties of the metal layer. Furthermore, under ambient conditions there is always a certain concentration of water molecules, and hence an understanding of the water–metal layer interaction is of interest from the point of view of future application of these junctions as electronic devices.

There have been numerous studies addressing the properties of water–metal interfaces, both from an experimental as well as from a theoretical point of view [13–21], but there are still questions remaining. For example, it is not clear whether water at close-packed metal surfaces is crystalline or liquid at room temperature [19]. Again, progress in the clarification of structure benefits from a close collaboration between experiment and theory [22–24].

According to DFT calculations, the interaction between water and flat metal surfaces is relatively weak [16,18]. For example, the energy gain upon the adsorption of a H_2_O monomer on Pd(111) is about −0.33 eV [18]. Single H_2_O molecules on metal surfaces preferentially occupy top site positions creating a one-fold oxygen–metal bond, with O–H bonds oriented parallel to the surface [25]. Layers of water on (111) metal surfaces are traditionally assumed to be arranged in an ice-like hexagonal bilayer structure with every second water molecule bound to the metal surface via the oxygen atom. The other water molecules have one hydrogen atom either pointing away from the surface (H_up_) or towards the surface (H_down_). In such an arrangement the adsorption energy related to one H_2_O in the gas phase is higher compared to the adsorption energy of a single water molecule, e.g., for the H_down_ structure on Pd(111) it is −0.56 eV per molecule. However, the dominating contribution is coming from intermolecular hydrogen bonds rather then from water–molecule interactions [16,18]. Consequently, because of the rather weak metal–water interaction the electronic structure of the metal substrate remains almost unaffected upon the water adsorption [26]. On the other hand, the adsorbed water bilayers become strongly polarized which leads to a significant work function change upon water adsorption on more strongly interacting transition metal surfaces such as, e.g., Pd/Au(111) [19].

In this paper, we use periodic DFT calculations to focus on the interaction of water molecules with the palladium monolayer prepared on a 4-mercaptopyridine SAM on Au(111), forming a Au/Mpy/Pd/H_2_O complex. We determine the stability of an isolated water molecule, as well as of a water layer arranged in a hexagonal bilayer, at the preferential adsorption sites on the densely packed palladium monolayer of the Au/Mpy/Pd system. In addition, we concentrate on the structural and electronic modification of the Au/Mpy/Pd complex upon water adsorption. In particular we will discuss the character of the palladium local density of states (LDOS) in the presence of water and compare the findings with experimental UPS spectra of the corresponding system.

## Results and Discussion

Before addressing the water adsorption on the Au/Mpy/Pd junction, we will first briefly discuss the structural details of the bare (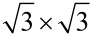)R30° Au/Mpy/Pd complex. This structure was adopted as the initial configuration for all geometry optimizations of the complexes with water. In this structure, there is one Mpy molecule and three metal atoms per layer in the unit cell. Mpy molecules are bound to the gold substrate via a two-fold S–Au bond at the near-bridge fcc site, which was previously determined as the most stable site of the molecule in the (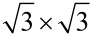)R30° structure on the Au(111) surface [27]. Note that the plane of the Mpy aromatic ring is tilted by 34° with respect to the Au(111) surface normal. The connection between the Mpy molecule and the densely packed palladium monolayer is realized via a one-fold N–Pd bond. In such an arrangement, one palladium atom is located directly above the nitrogen atom with a N–Pd distance of ~2.09 Å whereas the other two palladium atoms in each unit cell do not directly interact with the SAM. In the following, the two Pd species are distinguished with the former type denoted by Pd_b_ and the latter by Pd_n_, respectively.

We will first consider a single H_2_O molecule within the (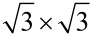)R30° unit cell to elucidate the interaction between a water monomer and the palladium layer of the Au/Mpy/Pd complex. This corresponds to a water coverage (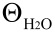) of 1/3 of a monolayer (ML) in which individual H_2_O molecules are relatively isolated from each other and do not form intermolecular hydrogen bonds. In the second step we add another H_2_O molecule to the layer thus increasing 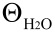 to 2/3 ML. As a consequence of the higher density, the water molecules form a hydrogen-bonded ice-like bilayer structure which is well-known from theoretical studies of water layers on close-packed hexagonal transition metal surfaces [18–19]. In this structure, every second H_2_O molecule is in a parallel configuration with respect to the metal surface, forming bonds via the oxygen atom to one metal atom, while the other set of H_2_O molecules are oriented with one hydrogen atom pointing either down or up, depending on the specific metal substrate.

### Structure of water on the Au/Mpy/Pd junction

Two types of water orientation were considered as the starting geometry of the structure optimization of a single H_2_O molecule on the Au/Mpy/Pd surface. First, we set the initial condition for the adsorption geometry of a H_2_O monomer on bulk Pd(111), in which the oxygen atom is at the top site 2.28 Å above the surface with the O–H bonds oriented parallel to the surface [18]. The top site of both types of palladium atoms Pd_b_ and Pd_n_ was considered as the starting adsorption position. In addition, an initial water structure with one O–H bond oriented towards a palladium atom (H_down_ structure) was also used in the structure optimization since this structural motive is present in water bilayers on metal surfaces [18–19].

**Figure 1:**
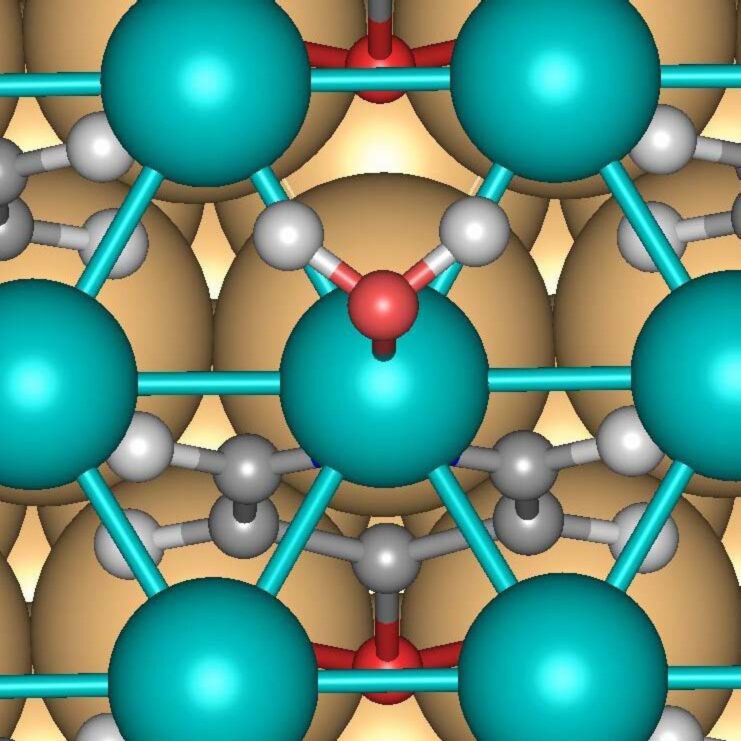
Top view of the optimized structure of a single H_2_O molecule on the palladium monolayer of the (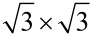)R30° Au/Mpy/Pd complex.

Only one stable position with an isolated H_2_O molecule located at the top site of the Pd_b_ atom was found within the Au/Mpy/Pd/H_2_O complex. The optimized structure is depicted in Figure 1. The geometry parameters of the most stable configurations together with the corresponding adsorption energies are listed in Table 1. The energy gain (−*E*_ads_) upon adsorption of a single H_2_O molecule on the bare Au/Mpy/Pd system is about 1.060 eV indicating a rather strong interaction in contrast to the relatively weak interaction between H_2_O and the (111) surfaces of transition metals [18–19]. Compared to water on bulk Pd(111), the O–Pd bond is shorter by about 0.12 Å. Interestingly, in this adsorption configuration the Pd_b_ atom is involved in two covalent bonds, to the H_2_O molecule on the upper side through an O–Pd bond and to the Mpy-SAM through a N–Pd bond on the bottom side. Usually one would assume that the Pd atom that does not participate in the bonding to the SAM would show the stronger binding to additional adsorbates. Note that the N–Pd bond is only negligibly shortened with respect to the situation in the bare Au/Mpy/Pd complex. The water molecule assumes a flat configuration with the O–H bonds oriented parallel to the surface. The O–H bond is only slightly elongated by 0.02 Å and the H–O–H angle is negligibly reduced by 1.4° with respect to that for H_2_O in the gas phase.

**Table 1:** Optimized geometry parameters and adsorption energies of a H_2_O monomer and a H_2_O hexagonal bilayer on a Au/Mpy/Pd contact within a (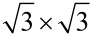)R30° geometry. The O–Pd_b_ value refers to the water molecule bound via the O atom to Pd, and the H–Pd_n_ distance is given for the H_down_ atom in the water bilayer. *E*_ads_ is the energy gain per H_2_O molecule upon adsorption with respect to a H_2_O molecule in the gas phase.

	distances Å			*E*_ads_ (eV)
	N–Pd_b_	O–Pd_b_	H–Pd_n_	

Au/Mpy/Pd	2.09			
Au/Mpy/Pd/H_2_O	2.01	2.12		−1.060
Au/Mpy/Pd/( 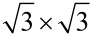 )H_2_O	2.02	2.14	1.96	−0.837

In order to check whether this is a consequence of the lowered coordination of the Pd atoms in the monolayer, or of the changes induced by the N–Pd_b_ interaction, we considered a free-standing palladium (111) monolayer using a (3 × 3) unit cell. Within this model, we first calculated the interaction of an isolated H_2_O molecule with the bare monolayer (Pd_monolayer_/H_2_O), i.e., without any attached Mpy molecule. Interestingly enough, we obtained an adsorption energy of −0.34 eV with an O–Pd bond distance of 2.28 Å, which is similar to the situation for H_2_O/Pd_bulk_ (111) [18], and this means that the water bonding to a free Pd(111) layer is weaker than that to a Pd layer deposited on the Mpy-SAM.

We extended the model by considering the additional adsorption of pyridine (Pyr) molecules on the other side of the Pd layer. This was motivated by the assumption that Pyr binds to Pd in the same way as Mpy since the sulfur head group of Mpy hardly affects the N–Pd contact. Upon the attachment of an up-right standing pyridine molecule to the Pd monolayer, with the water molecule adsorbed at the other side, the water adsorption energy was lowered to *E*_ads_ = −1.10 eV, i.e., the water binding became stronger, and the O–Pd_b_ distance decreased to 2.11 Å. Note that the optimized N–Pd bond length in this configuration is about 2.03 Å. This means that indeed the presence of a N–Pd_b_ bond leads to a stronger binding of water to the same Pd_b_ atom. The isolated H_2_O molecule was also placed on top of the Pd_n_ atom neighboring the Pd_b_ atom that was involved in the interaction with the pyridine molecule. Surprisingly, this structure turned out to be unstable, and not even meta-stable, because of the strong attraction of the water molecule to the Pd_b_ atom.

**Figure 2:**
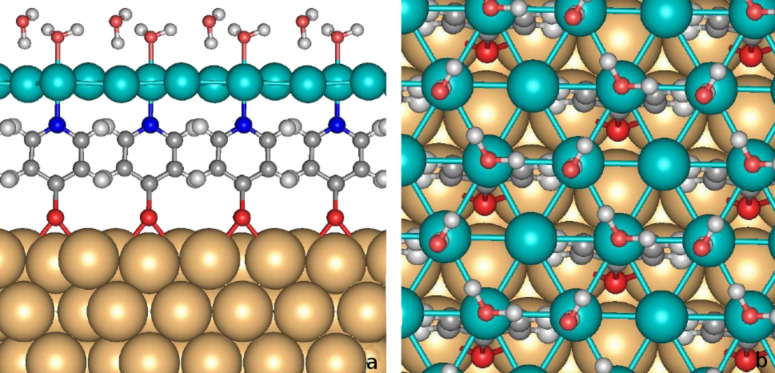
Side (a) and top (b) view of the optimized structure of water hexagonal bilayer on the palladium monolayer of (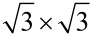)R30° Au/Mpy/Pd complex.

In the next step we added a second water molecule on top of the Pd layer of the (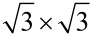)R30° Au/Mpy/Pd structure to complete the ice-like water bilayer (H_2_O_hex_) and examined the interaction between this water bilayer and the Au/Mpy/Pd contact. The optimized geometry of this system is illustrated in Figure 2. There are two sets of H_2_O molecules within the bilayer. In the optimized structure the first type of water molecule (H_2_O_O–Pd_) is located at the top site above the Pd_b_ atoms in a geometry similar to the one of a single H_2_O molecule on Au/Mpy/Pd (Table 1). Both H atoms of this H_2_O_O–Pd_ molecule are involved in hydrogen bonds (H-bond) to two water molecules of the second type. In those H_2_O molecules, only one hydrogen atom forms a H-bond to one H_2_O_O–Pd_ molecule, whereas the second H atom is directed towards one Pd_n_ atom with a Pd–H distance of 1.96 Å, corresponding to the H_down_ structure. Surprisingly, the H_up_ configuration is not stable on the Au/Mpy/Pd system. Consequently, there are three inequivalent Pd atoms within the monolayer: (i) The Pd_b_ atom directly interacting with the O atom of the H_2_O molecule and the N atom of the Mpy molecule, respectively, (ii) the Pd_n_ atom interacting with the H atom of the second H_2_O molecule, and (iii) the noninteracting Pd_n_ atom located in the middle of the hexagonal ring of the water bilayer.

The adsorption of H_2_O molecules forming a H_2_O_hex_ water bilayer on the Au/Mpy/Pd junction is less favorable than the adsorption of an isolated H_2_O monomer (Table 1), by about ~0.22 eV per molecule. This is surprising since usually the attractive water–water interaction through intermolecular hydrogen bonds contributes significantly to the stability of water layers on metal surfaces [16,18]. However, it has to be noted that there is no way to uniquely decompose the two contributions to the water adsorption energy since the strengths of the water–metal and the water–water bonding are not independent of one another [16]. Still, qualitative trends can be deduced when the adsorption energy of the water bilayer on the Au/Mpy/Pd complex is compared with the energy gain upon the formation of a free-standing water bilayer (i.e., without a metal substrate) in the geometry of the adsorbed H_2_O_hex_ bilayer on the Au/Mpy/Pd complex. Note that the latter energy contribution is entirely due to the H-bond formation.

The energy gain upon the assembly of a free-standing relaxed water bilayer, within the used unit cell, amounts to ~0.37 eV per H_2_O molecule. Using the geometry of the H_2_O_hex_ bilayer on the Au/Mpy/Pd complex reduces the energy gain to ~0.20 eV as a consequence of the significant internal reorganization of the molecules in the bilayer upon the interaction with the Pd surface. Furthermore, in the bilayer not all water molecules are adsorbed in the optimal configuration as far as the water–metal bond is concerned, since only every second water molecules is bound via the oxygen atom to the metal. These two effects, reduced water–water attraction and non-optimal water adsorption configuration, together with a strong, dominating water metal bond, make the adsorption of isolated molecules energetically more favorable than the adsorption of the water bilayer, in contrast to bulk metal surfaces where the major contribution to *E*_ads_ appears to come from the intermolecular H-bonds [16,18].

Note furthermore that using a (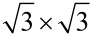)R30° periodicity implies rather strict boundary conditions to the possible structures, e.g., it favors a hexagonal symmetry of the H_2_O layers. In order to estimate the consequences of these geometry restrictions, we additionally considered a Pd monolayer using a (3 × 3) unit cell with three pyridine molecules placed at the positions corresponding to the (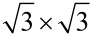)R30° structure, i.e., we considered a (3 × 3) Pyr/Pd_monolayer_/H_2_O_hex_ complex. By removing the pyridine molecules, the interaction between a pure Pd_monolayer_ and a water bilayer was also examined.

We found no difference in the structural and energy parameters between the Pyr/Pd_monolayer_/H_2_O_hex_ and the Au/Mpy/Pd/H_2_O_hex_ systems with respect to the water structure, i.e., *E*_ads_ = −0.85 eV and the O–Pd and H–Pd distances of 2.14 and 1.93 Å, respectively, remained basically unchanged. The H-bond contribution to *E*_ads_ is about −0.22 eV, similar to that in the (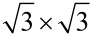)R30° unit cell. Upon removal of the Pyr molecules the H_2_O layer became significantly relaxed. The O–Pd distance increased to 2.35 Å, but the Pd–H bond of 1.97 Å became only slightly elongated. Correspondingly, *E*_ads_ decreased to −0.51 eV, but, the energy of the H-bonds only changed by a small amount to −0.29 eV. Note that within the Pd_monolayer_ /H_2_O_hex_ structure the H_up_ water bilayer configuration turns out to be a local minimum, i.e., it becomes meta-stable, but it is still about 0.3 eV less stable than the H_down_ arrangement.

Apparently, the stronger binding between the water molecules and the palladium monolayer deposited on top of the SAM compared to water on bulk metal substrates is due to the presence of the Mpy molecules binding to Pd from the bottom side. The fact that the palladium atoms in the (111) monolayer are less coordinated than the Pd atoms in a (111) surface apparently plays a minor role for the stability in the water complex. Ab initio molecular dynamics simulations showed that at room temperature the hexagonal water bilayer structure on bulk metal surfaces becomes disordered [19], but it may persist on the Au/Mpy/Pd junction because of the higher stability of the H_2_O layer, which is not governed by intermolecular H-bond interactions. Still, it could strongly depend on the structure of the molecules in the SAM on which the Pd layer is deposited.

**Figure 3:**
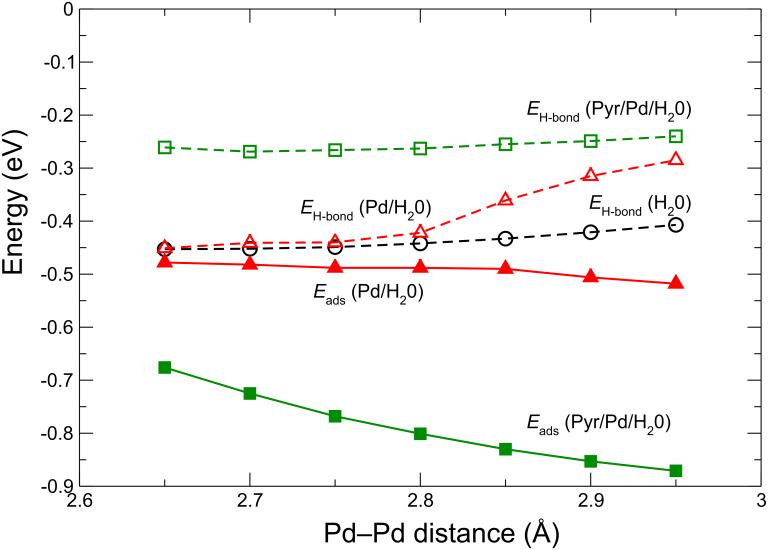
Adsorption energies *E*_ads_ of the water bilayer together with the contribution originating entirely from H-bonds *E*_H–bond_ obtained within a (3 × 3) unit cell as a function of the Pd–Pd distance from 2.65 Å to 2.95 Å, which are the nearest-neighbor distances in a free Pd monolayer and in bulk Au, respectively. The latter value has been used in the calculations of the full junction. Water in the bilayer structure is adsorbed on the (3 × 3) Pd monolayer without (Pd/H_2_O) and in the presence of pyridine molecules attached to the bottom side of the Pd layer (Pyr/Pd/H_2_O). The H-bond energy contribution of a free-standing H_2_O bilayer (H_2_O) without Pd was added as a reference.

Note that so far we have only considered situations in which the lateral lattice constant of the Pd layer is dictated by the periodicity of the Au(111) substrate. However, it is fair to say that the Pd–Pd distance in the real system is not known, because in the scanning tunneling microscopy (STM) measurements the lateral distances could not be exactly calibrated [8]. It might well be that the Pd layer is not commensurate with the Au(111) substrate. A modified Pd–Pd spacing would of course influence the strength of the O–Pd bonds [28–30] and the H-bonds within the bilayer and thus affect the stability of the H_2_O layer on the Pd monolayer. In order to check the effect of varying the Pd–Pd distance on the stability of the H_2_O/Pd complex we changed the lateral constant of the (3 × 3) structure in a systematic fashion to cover Pd–Pd distances from 2.65 Å to 2.95 Å. The lower limit with a Pd–Pd distance of 2.65 Å corresponds to that for the optimized free-standing Pd monolayer [8], whereas the upper limit of 2.95 Å is the nearest-neighbor distance in bulk Au, which has been used in the calculations of the whole junction. The total adsorption energies together with the energy contribution coming from the H-bonds for the H_2_O bilayer, either (i) free-standing (without a Pd monolayer), or (ii) interacting with the bare Pd monolayer, or (iii) interacting with the Pyr/Pd complex, are plotted in Figure 3.

The water adsorption energy *E*_ads_ in a bilayer on the Pyr/Pd_monolayer_ complex decreases slightly with decreasing Pd–Pd distance, by about 0.06 eV per 0.1 Å. At the same time, the stabilization energy of the free-standing H_2_O bilayer coming from the H-bonds increases by only about 0.015 eV per 0.1 Å, thus remaining almost constant in the range of the considered lattice constants.

Hence it is not surprising that in the Pyr/Pd_monolayer_/H_2_O_hex_ system the energy contribution coming from H-bonds remains practically constant, being about 0.2 eV smaller than the stabilization energy of the pure H_2_O bilayer. This means that a change of *E*_ads_ upon a variation of the Pd–Pd lattice spacing is almost entirely due to the modification of the O–Pd interaction strength. Since this dependence is also rather weak, there should only be a small influence of the Pd lattice spacing on the stability of the water bilayer on the Au/Mpy/Pd junction.

### Electronic properties of the Au/Mpy/Pd/H_2_O complex

**Figure 4:**
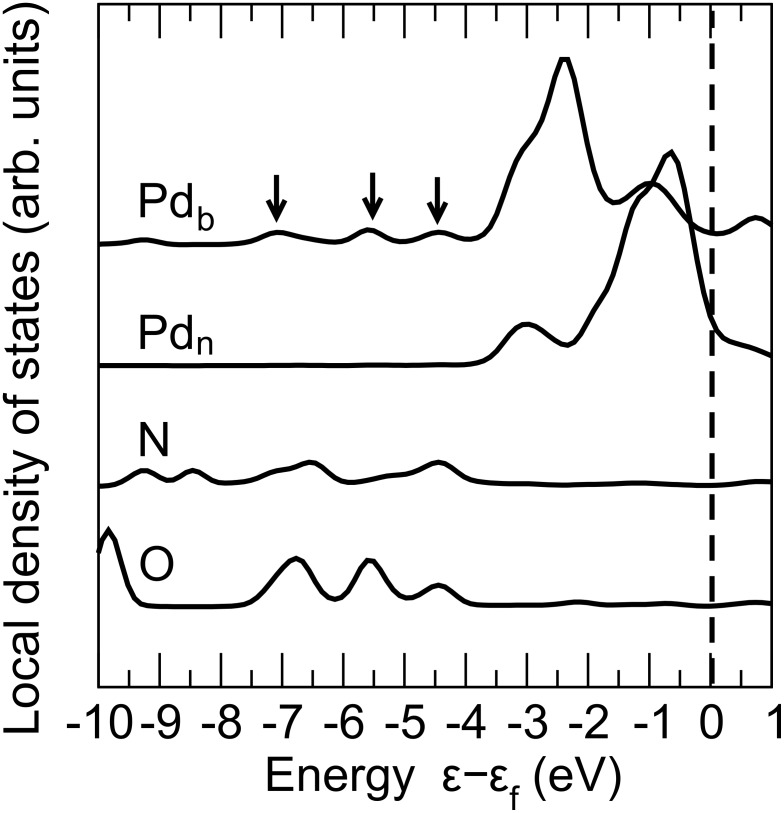
Local density of states (LDOS) of the Au/Mpy/Pd/H_2_O complex at a water coverage of 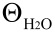 = 1/3 ML. Plotted, from the top, is the LDOS of the Pd_b_ atom bound to the nitrogen atom of 4-mercaptopyridine and the oxygen atom of the water molecule, of the Pd_n_ atom not interacting with any molecules, of the nitrogen atom (N) of the 4-mercaptopyridine molecule, and of the oxygen atom of the water molecule (O). The bonding states between Pd_b_, N, and O atoms are marked with arrows.

The stabilization of the water adsorption on the Pd layer by the interaction with the underlying SAM is a rather surprising result, because usually one would assume that a higher coordination of the Pd atoms would lead to a smaller binding strength. In order to elucidate the nature of the N_Mpy_–Pd_b_–O_w_ bonding and its effect on the electronic structure of the Au/Mpy/Pd junction we determined the local density of states (LDOS) of the species involved in the complex formation, namely nitrogen, Mpy, oxygen, and palladium. The spectra of the various atoms are plotted in Figure 4.

As evident from Figure 4, the electronic structure of the Pd_b_ atom is considerably modified upon the formation of the N–Pd–O bonding complex compared to the LDOS of the non-interacting Pd_n_. The latter LDOS is in fact rather close to that of a bare Pd monolayer (e.g., [8]). The Pd_b_ atom exhibits a significant reduction of the LDOS near the Fermi energy, whereas the small change of the LDOS of the Pd_n_ atom indicates that the effect of the N_Mpy_–Pd_b_–O_w_ bond is rather localized.

Figure 4 indicates furthermore that there is a hybridization between N_Mpy_, O_w_, and Pd_b_ states (marked by the arrows in Figure 4) leading to three separated peaks localized at −4.46, −5.56, and −6.98 eV below *E*_f_. As a further consequence, the Pd_b_ LDOS close to the Fermi level is reduced, and the maximum of the Pd_b_ LDOS is shifted to about −2.35 eV below *E*_f_.

**Figure 5:**
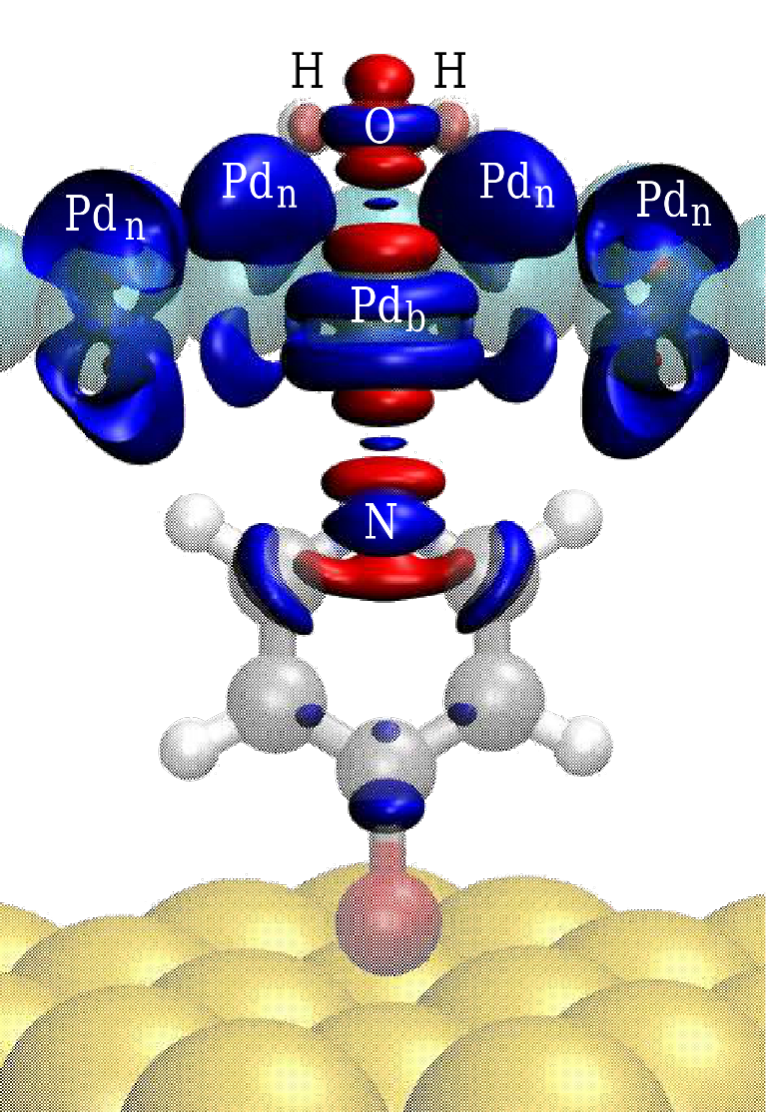
Charge density difference in an isodensity representation calculated as the difference between the charge density of the Au/Mpy/Pd/H_2_O complex and the sum of the charge densities of the Au/Mpy, Pd, and H_2_O subsystems. Blue and red surfaces depict the region of charge accumulation and depletion, respectively. The selected surfaces correspond to the charge isodensity of 0.016 *e*/Å^3^ and −0.044 *e*/Å^3^ encapsulating the total charge of 0.44 and −0.44 *e*, respectively.

Such a strong change of the density of states also indicates a substantial charge transfer between the involved constituents. This is illustrated by the charge density difference isodensity surfaces shown in Figure 5, which correspond to the difference between the charge density of the interacting Au/Mpy/Pd/H_2_O complex and the sum of the charge densities of the isolated Au/Mpy, Pd, and H_2_O subsystems in the same configuration. There is a strong charge rearrangement along both the N–Pd_b_ and Pd_b_–O bonds indicative of the covalent character of the bonds. The calculated patterns suggest a hybridization between the p*_z_* orbitals of N_Mpy_ and O_w_, and the 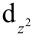 orbital of the Pd_b_ atoms upon the formation of the N_Mpy_–Pd_b_–O contact with *z* being the coordinate along the surface normal. However, the regions of the charge depletion are relatively localized in the region of the covalent bonds, and Figure 5 also reveals a diffuse charge accumulation around the non-bonding Pd_n_ atoms.

In order to get a more quantitative picture of the charge redistribution within the molecule/metal complex, we performed a Bader analysis [31–32]. To estimate the influence of water on the charge transfer we compare the results of the system without water (Au/Mpy/Pd) with the results for the system with adsorbed water (Au/Mpy/Pd/H_2_O). The results are summarized in Table 2.

**Table 2:** Bader analysis of the total electronic charge depletion/accumulation in the Au slab, the 4-mercaptopyridine molecule, the palladium layer (in parenthesis: Charge localized on the Pd_b_ atom only), and the water layer (H_2_O) within the Au/Mpy/Pd, Au/Mpy/Pd/H_2_O, and Au/Mpy/Pd/(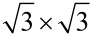)H_2_O complexes. The partial charge excess/deficiency, in electrons (*e*), is defined relative to the uncharged subsystems.

	Au	Mpy	Pd (Pd_b_)	H_2_O

Au/Mpy/Pd	+0.167	−0.230	+0.062 (+0.232)	
Au/Mpy/Pd/H_2_O	+0.161	−0.268	+0.075 (+0.359)	+0.032
Au/Mpy/Pd/( 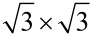 )H_2_O	+0.158	−0.272	+0.130 (+0.354)	−0.016

In the bare Au/Mpy/Pd system without water adsorption, the Mpy molecular layer sandwiched between the Au substrate and the Pd monolayer becomes negatively charged. Consequently, there is an electron deficiency at both metal electrodes. This suggests a substantial polarization at both interfaces. The electrons are transferred from Au to Mpy along the two-fold coordinated S–Au bonds. This is completed by the additional electron transfer from Pd to Mpy via a single N–Pd_b_ bond. Closer inspection of the charge distribution within the Pd layer (note that the charge of the Pd_b_ atom is listed in parentheses in Table 2) reveals a substantial redistribution of the electron density between the Pd_b_ and Pd_n_ atoms. Although there is a charge deficiency of about 0.232 *e* on the Pd_b_ atom, the electron density on the two Pd_n_ atoms per unit cell is increased by 0.17 *e* clearly indicating an electron transfer from Pd_b_ to Pd_n_.

Upon the adsorption of a single H_2_O molecule on the Pd layer, the accumulation of electrons at the Mpy molecule further increases, but the electron depletion at the Au electrode remains practically unaltered. This means that the S–Au bond is hardly affected by the adsorption of water on the palladium layer. This is also reflected in the length of the S–Au bond, which does not change upon the water adsorption, remaining at 2.57 Å. The electron gain of Mpy due to the H_2_O→Pd→Mpy charge transfer is accompanied by charge depletion on the H_2_O molecule and a further polarization within the Pd layer through charge transfer from Pd_b_ to Pd_n_. This inner polarization explains why a single H_2_O molecule does not form a (meta-)stable structure on-top of the Pd_n_ atoms. The higher electron density at the Pd_n_ atoms increases the Pauli repulsion between the electron clouds of the closed-shell H_2_O molecule and the Pd_n_ atoms. As a consequence, Pd_n_ atoms would not be covered by water molecules at low water coverage.

Although the character of the interaction between the oxygen atom of the H_2_O molecule and the Pd_n_ atom is repulsive, the interaction between the hydrogen atom of the H_2_O molecule and the Pd_n_ atom must be attractive since the H_2_O bilayer on Au/Mpy/Pd is preferentially oriented in the H_down_ configuration with the hydrogen atoms pointing towards the Pd_b_ atoms thus forming Pd_n_–H bonds. Since the electron screening of the hydrogen nucleus in the H_2_O molecule is partially weakened due to the polarization of the O–H bond, the hydrogen atom can then bind to the additional electrons on the Pd_n_ atom. The electron density in fact shifts from the Pd_n_ atom to the H atoms of the water molecule, which is suggested by the increased electron deficiency within the whole Pd layer upon the deposition of the water bilayer. The charge at the Pd_b_ atom, however, remains the same compared to the case of the adsorption of a single water molecule on the Au/Mpy/Pd junction.

**Figure 6:**
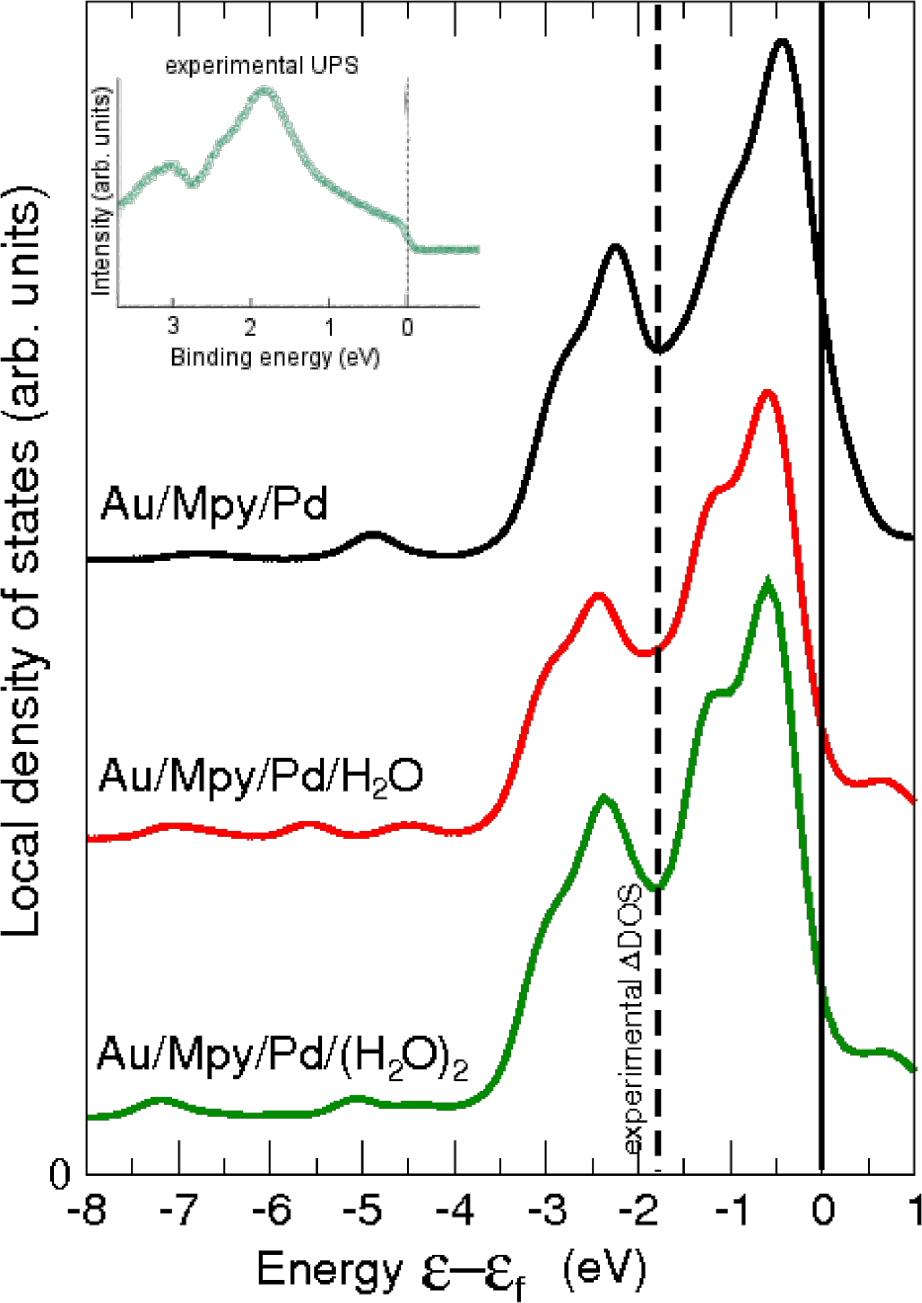
Local density of states of the Pd monolayer of the Au/Mpy/Pd system with and without water. Au/Mpy/Pd denotes bare the system without water, Au/Mpy/Pd/H_2_O denotes an isolated H_2_O molecule on the surface (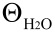 = 1/3 ML), and Au/Mpy/Pd/(H_2_O)_2_ denotes a water hexagonal bilayer attached on the surface (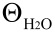 = 2/3 ML). In the inset, the experimental UPS spectrum of the corresponding system is included (adopted from [8]).

Finally, we compare the calculated total DOS of the palladium monolayer in the presence of various amounts of water with the experimental UPS spectrum of the palladium layer in the Au/Mpy/Pd system [8]. The DOS of the Pd monolayer in the Au/Mpy/Pd junction with 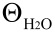 = 0, 1/3 ML (isolated H_2_O), and 2/3 ML (water bilayer) is plotted in Figure 6 together with the experimental spectrum adopted from [8]. The theoretical results should be compared to the calculated LDOS of the free-standing Pd monolayer plotted in [8]. The results can be summarized as follows: (i) Despite the strong interaction between water and the Au/Mpy/Pd complex, the presence of water induces only a negligible modification of the Pd LDOS compared to the bare Au/Mpy/Pd model; (ii) The LDOS of the Pd layer with the two different water coverages is basically equivalent; (iii) In strong contrast to the experimental spectrum none of the calculated LDOS shows a considerable reduction of the DOS near the Fermi energy.

It is in fact unsurprising that the LDOS of the Pd layer with the isolated H_2_O molecule and with the H_2_O bilayer structure are so similar, as the residual bond between the H atom of the H_2_O molecule and the Pd atom is rather weak. Concerning the overall character of the spectra, qualitatively these are convoluted from the contributions of Pd_n_ and Pd_b_ atoms, which are present in the monolayer in a ratio of 2/1. This means that the Pd_n_ atoms which exhibit only a small downshift of the LDOS dominate the DOS of the whole Pd monolayer independent whether there is only a N–Pd_b_ bond (in the system without water) or a N–Pd_b_–O bond (in the presence of water) since both bonding types affect the Pd monolayer only locally. Consequently, the adsorption of water cannot explain the observed downshift of the Pd DOS.

On the other hand, the rather stable water structures on the Au/Mpy/Pd junction might have a considerable impact on the adsorption of other species and directly influence the concentration of adsorbates on the Pd surface in equilibrium. As an alternative explanation, the adsorption of hydrogen atoms on the Pd layer might explain the observed UPS spectra since the presence of hydrogen on a Au/SAM/Pd junction can change the electronic structure of the Pd layer rather significantly, as shown in the case of the Au/ATP/Pd complex [12]. Work along this line is in progress.

## Conclusions

We have investigated the geometric and electronic structure of a Au/Mpy/Pd junction upon the adsorption of water by first principles electronic structure calculations based on density functional theory. An isolated water molecule on the palladium monolayer of the Au/Mpy/Pd junction forms a relatively stable complex bonded through the oxygen atom of water to a single palladium atom. This bond is in fact stabilized through the N–Pd bond of the Pd layer with the underlying SAM leading to a much higher water adsorption energy on the Pd monolayer compared to bulk Pd(111). This stabilization is also present in an ice-like hexagonal water bilayer adsorbed in a (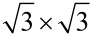)R30° structure on the Au/Mpy/Pd junction. This is caused by a strong polarization within the Pd monolayer induced by the Pd–N bond.

The formation of the N–Pd–O complex causes a significant modification of the local density of states of the Pd atom involved in this complex, resulting in a large decrease of the LDOS at the Fermi level. On the other hand, the LDOS of the other Pd atoms not interacting with the Mpy and the H_2_O molecules is hardly changed. The overall DOS of the Pd layer is only weakly modified by the adsorption of water which thus can not explain the experimentally observed strong reduction of the DOS of the Pd layer in the junction at the Fermi energy.

## Experimental

Self-consistent periodic DFT calculations were performed employing the Vienna ab initio simulation package (VASP) [33]. The exchange-correlation effects were described within the generalized gradient approximation using the Perdew–Burke–Ernzerhof (PBE) functional [34]. The interaction of the electrons with the ionic cores was treated with the projected augmented wave (PAW) method [35–36], and the Kohn–Sham one-electron valence states were expanded in a basis of plane waves with a cutoff energy of 400 eV. All calculations were spin-polarized since palladium in low-dimensional structures can become magnetic [37].

Most of the calculations are done for a (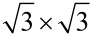) surface unit cell. Within the supercell approach the Au(111) substrate was represented by slabs of five-layers, in which the two topmost layers were relaxed during the geometry optimization, while the rest of the gold atoms were kept fixed at the positions corresponding to the bulk Au crystal. The Au lattice spacing [*d*(Au–Au) = 2.95Å] was adopted from the equilibrium geometry of bulk Au calculated at the same level of the theory. To separate the Au slabs in the non-periodic direction along the surface normal a vacuum region of thickness 28 Å was inserted into the supercell. To carry out the Brillouin-zone integration, a Monkhorst–Pack [38] of 11 × 11 × 1 k-points were used.

The local density of states (LDOS) was calculated in order to interpret the experimental UPS spectra [8] of the palladium layer. To compare the experiment with the theoretical results we convoluted the calculated LDOS with a Gaussian of width 0.2 eV, thus taking into account the finite energy resolution of the experimental spectra as well as the generally observed broadening of spectroscopic features due to the finite life time of the photoionized states.

## References

[1] Baunach T, Ivanova V, Scherson D A, Kolb D M (2004). Langmuir.

[2] Manolova M, Boyen H-G, Kučera J, Groß A, Romanyuk A, Oelhafen P, Ivanova V, Kolb D M (2009). Adv Mater.

[3] Eberle F, Kayser M, Kolb D M, Saitner M, Boyen H-G, D’Olieslaeger M, Mayer D, Wirth A (2010). Langmuir.

[4] Eberle F, Metzler M, Kolb D M, Saitner M, Wagner P, Boyen H-G (2010). ChemPhysChem.

[5] Manolova M, Ivanova V, Kolb D M, Boyen H-G, Ziemann P, Büttner M, Romanyuk A, Oelhafen P (2005). Surf Sci.

[6] Manolova M, Kayser M, Kolb D M, Boyen H-G, Ziemann P, Mayer D, Wirth A (2007). Electrochim Acta.

[7] Eberle F, Saitner M, Boyen H-G, Kučera J, Groß A, Romanyuk A, Oelhafen P, D’Olieslaeger M, Manolova M, Kolb D M (2010). Angew Chem, Int Ed.

[8] Boyen H-G, Ziemann P, Wiedwald U, Ivanova V, Kolb D M, Sakong S, Groß A, Romanyuk A, Büttner M, Oelhafen P (2006). Nat Mater.

[9] Ulusoy I S, Scribano Y, Benoit D M, Tschetschetkin A, Maurer N, Koslowski B, Ziemann P (2011). Phys Chem Chem Phys.

[10] Keith J A, Jacob T (2010). Electrochim Acta.

[11] Keith J A, Jacob T (2010). Chem–Eur J.

[12] Kučera J, Groß A (2010). Phys Chem Chem Phys.

[13] Henderson M A (2002). Surf Sci Rep.

[14] Feibelman P J (2002). Science.

[15] Meng S, Xu L F, Wang E G, Gao S W (2002). Phys Rev Lett.

[16] Roudgar A, Groß A (2005). Chem Phys Lett.

[17] Roudgar A, Groß A (2005). Surf Sci.

[18] Michaelides A (2006). Appl Phys A.

[19] Schnur S, Groß A (2009). New J Phys.

[20] Carrasco J, Santra B, Klimeš J, Michaelides A (2011). Phys Rev Lett.

[21] Schnur S, Groß A (2011). Catal Today.

[22] Cerdá J, Michaelides A, Bocquet M-L, Feibelman P J, Mitsui T, Rose M, Fomin E, Salmeron M (2004). Phys Rev Lett.

[23] Tatarkhanov M, Ogletree D, Rose F, Mitsui T, Fomin E, Maier S, Rose M, Cerdá J, Salmeron M (2009). J Am Chem Soc.

[24] Nie S, Feibelman P, Bartelt N, Thürmer K (2010). Phys Rev Lett.

[25] Michaelides A, Ranea V A, de Andres P L, King D A (2003). Phys Rev Lett.

[26] Gohda Y, Schnur S, Groß A (2009). Faraday Discuss.

[27] Kučera J, Groß A (2008). Langmuir.

[28] Mavrikakis M, Hammer B, Nørskov J K (1998). Phys Rev Lett.

[29] Groß A (2006). Top Catal.

[30] Groß A (2009). J Phys: Condens Matter.

[31] Bader R F W (1990). Atoms in Molecules - A Quantum Theory.

[32] Henkelman G, Arnaldsson A H, Jónsson H (2006). Comput Mater Sci.

[33] Kresse G, Furthmüller J (1996). Phys Rev B.

[34] Perdew J P, Burke K, Ernzerhof M (1996). Phys Rev Lett.

[35] Blöchl P E (1994). Phys Rev B.

[36] Kresse G, Joubert D (1999). Phys Rev B.

[37] Groß A (2009). Theoretical surface science – A microscopic perspective.

[38] Monkhorst H J, Pack J D (1976). Phys Rev B.

